# The Peripheral Blood Transcriptome Identifies the Presence and Extent of Disease in Idiopathic Pulmonary Fibrosis

**DOI:** 10.1371/journal.pone.0037708

**Published:** 2012-06-22

**Authors:** Ivana V. Yang, Leah G. Luna, Jennifer Cotter, Janet Talbert, Sonia M. Leach, Raven Kidd, Julia Turner, Nathan Kummer, Dolly Kervitsky, Kevin K. Brown, Kathy Boon, Marvin I. Schwarz, David A. Schwartz, Mark P. Steele

**Affiliations:** 1 Center for Genes, Environment and Health, National Jewish Health, Denver, Colorado, United States of America; 2 Pulmonary, Allergy, Critical Care Medicine, Duke University Medical Center, Durham, North Carolina, United States of America; 3 Interstitial Lung Disease Laboratory, National Jewish Health, Denver, Colorado, United States of America; 4 Department of Medicine; University of Colorado, Denver, Aurora, Colorado, United States of America; 5 Excerpta Medica, Amsterdam, The Netherlands; 6 Department of Medicine, Vanderbilt University School of Medicine, Nashville, Tennessee, United States of America; I2MC INSERM UMR U1048, France

## Abstract

**Rationale:**

Peripheral blood biomarkers are needed to identify and determine the extent of idiopathic pulmonary fibrosis (IPF). Current physiologic and radiographic prognostic indicators diagnose IPF too late in the course of disease. We hypothesize that peripheral blood biomarkers will identify disease in its early stages, and facilitate monitoring for disease progression.

**Methods:**

Gene expression profiles of peripheral blood RNA from 130 IPF patients were collected on Agilent microarrays. Significance analysis of microarrays (SAM) with a false discovery rate (FDR) of 1% was utilized to identify genes that were differentially-expressed in samples categorized based on percent predicted D_L_CO and FVC.

**Main Measurements and Results:**

At 1% FDR, 1428 genes were differentially-expressed in mild IPF (D_L_CO >65%) compared to controls and 2790 transcripts were differentially- expressed in severe IPF (D_L_CO >35%) compared to controls. When categorized by percent predicted D_L_CO, SAM demonstrated 13 differentially-expressed transcripts between mild and severe IPF (< 5% FDR). These include CAMP, CEACAM6, CTSG, DEFA3 and A4, OLFM4, HLTF, PACSIN1, GABBR1, IGHM, and 3 unknown genes. Principal component analysis (PCA) was performed to determine outliers based on severity of disease, and demonstrated 1 mild case to be clinically misclassified as a severe case of IPF. No differentially-expressed transcripts were identified between mild and severe IPF when categorized by percent predicted FVC.

**Conclusions:**

These results demonstrate that the peripheral blood transcriptome has the potential to distinguish normal individuals from patients with IPF, as well as extent of disease when samples were classified by percent predicted D_L_CO, but not FVC.

## Introduction

Idiopathic Pulmonary Fibrosis (IPF) is categorized as an interstitial lung disease (ILD) and is the most common subtype of idiopathic interstitial pneumonias (IIP) comprising nearly 71% of the total cases [1]. Of the IIPs, IPF has the poorest prognosis with a 50% mortality rate 3 years following diagnosis [2]. Prognostic indicators of IPF include progressive deterioration of clinical symptoms such as dyspnea, pulmonary function, and extent of disease on high-resolution chest CT [3]–[7]. While dyspnea scores have been used as a predictor of survival in IPF patients [8], it remains an ambiguous prognostic indicator since it is highly subjective. Pulmonary function tests such as diffusing capacity for carbon monoxide (D_L_CO) and forced vital capacity (FVC) have been utilized as predictive indicators [9], [10]. Studies demonstrate that a D_L_CO of <35% or a decline in D_L_CO >15% within a year period are associated with an increased mortality. Similarly, a decline of >10% in FVC over a six month period also indicated an earlier mortality [8], [11]. The overall extent of fibrosis on high-resolution chest CT (HRCT) characterized by a honeycomb pattern and reticulation predict survival [12]. Randomized prospective controlled clinical trials in IPF have demonstrated significant differences in the rate of decline in FVC and D_L_CO among the placebo arms of the trials indicating there is substantial disease heterogeneity within IPF [13]. Current indicators of disease progression fail to capture the dynamic biology associated with IPF particularly patients at risk for acute exacerbations of IPF [14]. Biomarkers that measure disease stage and activity would be of benefit in understanding the effects of novel treatments, disease progression, and the design of clinical trials with homogenous placebo and treatment groups.

Rosas and coworkers observed a differential expression of MMP7, MMP1, MMP8, IGFBP1, and TNFRSF1A proteins in the peripheral blood between familial interstitial pulmonary fibrosis and controls [15]. However, the use of these biomarkers to differentiate disease severity or extent of disease within the IPF cohort was not addressed. We hypothesize that peripheral blood transcriptional profiles from patients with IPF would enable us to distinguish patients with IPF from controls, and mild from more advanced disease stage, and allow for monitoring of the progression of disease in either sporadic or familial IPF.

## Methods

### Study Populations

One hundred thirty peripheral blood RNA specimens were collected from individuals enrolled in either the Interstitial Lung Disease (ILD) or the Familial Pulmonary Fibrosis (FPF) Programs conducted at National Jewish Health and Duke University. Only one individual case per family was utilized from the FPF repository. Individual samples had a consensus diagnosis of probable or definite IPF, and this was based on the ATS/ERS/JRS/ALAT criteria [16]. Subjects were excluded from selection if they were current smokers, or currently treated with agents that could alter mRNA levels such as glucocorticoids, azathioprine, or other immunomodulators. One-hundred twenty three RNA samples passed quality assurance parameters after RNA extraction, probe synthesis, and hybridization for further analysis. 53 of these samples were from sporadic cases of IPF, and 70 samples were from familial IPF. Peripheral blood gene expression profiles were analyzed on groups of individuals based on disease severity. Two pulmonary function measurements, D_L_CO and FVC, were used to stratify the cases into severe and mild disease categories. Mild disease is defined as either percent predicted D_L_CO ≥65% (N = 16) or FVC ≥75% (N = 27). Severe disease is defined as either D_L_CO ≤35% (N = 15), FVC ≤50% (N = 13). All of these were also compared to age and gender matched non-diseased, healthy controls (N = 27). Eight patients categorized as severe were used in both the D_L_CO and FVC analysis, whereas the mild disease classified by either D_L_CO or FVC are all distinct cases. Healthy control subjects are family members who participated in screening for the presence of pulmonary fibrosis, and after evaluation of their medical history, lung function, and chest CT, they were found to have no evidence of lung disease. Individual institutional review boards approved this research. All participants in this study provided written IRB-approved informed consent.

### Pulmonary Function Testing

FEV1 and D_L_CO measurement were obtained according to American Thoracic Society standards and guidelines [17].

### Expression Profiling

#### Peripheral blood RNA isolation and purification

Peripheral blood samples were collected in PAXgene RNA tubes (PreAnalytiX, 762165). RNA extraction and purification was performed manually utilizing the PAXgene Blood RNA kit (PreAnalytiX, 762164) according to the manufacturer’s protocol.

#### Total RNA quantification and quality characterization

Quantification of total RNA was measured via the Nanodrop ND-1000 spectrophotometer (NanoDrop Technologies, Wilmington, DE). Quality of the RNA was assessed with a RNA 6000 NanoChip (Agilent, Palo Alto, CA) on the 2100 Bioanalyzer (Agilent, Palo Alto, CA) by ratio comparison of the 18 S and 28 S rRNA bands.

#### Microarrays

Agilent Whole Human Genome Oligonucleotide Microarrays (Agilent, Palo Alto, CA), were used to determine gene expression levels in peripheral blood. Total RNA was used as a template for synthesis of cDNA utilizing the One Color Low Input Agilent Quick Amp Labeling Kit utilizing the Spike-In Kit to provide positive controls. The Agilent one-color microarray based gene expression analysis was followed per manufacturer’s instructions, and passed Agilent’s quality control (QC).

#### Microarray data analysis

Analysis was performed utilizing the Multi-Experiment Viewer (MeV) software package [18]. Significance analysis of microarrays (SAM) with a false discovery rate (FDR) of 1% and 5%, assessed by performing 100 permutations, was performed within the program to identify genes that were differentially-expressed between IPF samples categorized by percent predicted D_L_CO and FVC. All IPF samples were compared to normal controls to identify differentially-expressed genes. Principal component analysis (PCA) was carried out on genes identified by SAM analyses to identify outliers, and gene-based hierarchical clustering was performed to identify relationships among differentially-expressed genes related to mild or severe disease categorized by D_L_CO. All microarray data is minimal information about a microarray experiment (MIAME) compliant, and raw data has been deposited into the GEO database (GSE33566).

#### Gene ontology and functional network analysis

Data were analyzed through the use of Ingenuity Pathways Analysis (Ingenuity Systems, www.ingenuity.com). The Canonical Pathways Analysis identified the pathways, and the significance of these was determined by Fischer’s exact test. Biomarker Analysis was employed to identify the most relevant molecular biomarker candidates.

#### Validation

Quantitative real-time PCR was utilized to confirm differential expression of genes discovered by microarray analysis with an ABI 7900 HT Fast Real-Time PCR Detection System (Applied Biosystems, Foster City, CA) using forty cycles of amplification. All assays were performed in duplicate and data were analyzed by the ΔΔCt method utilizing glyceraldehyde 3 phosphate dehydrogenase (GAPDH) as an endogenous control [19].

## Results

### Demographics and Disease Severity

Age, gender, and smoking history of the study population stratified into mild and severe disease groups based on pulmonary function status (Tables 1 and 2) show a mean FVC of 85% predicted and D_L_CO 77.1% predicted in the mild disease group, and a mean FVC 42.5% predicted and 27.5% predicted D_L_CO in the severe disease group. The mean age is similar between groups when classified by either % predicted FVC (P = 0.18), or % predicted D_L_CO (P = 0.87), but the mean age of either disease group is slightly older compared to controls (P = 0.15). The majority of patients are male in both mild and severe disease categories, and similar between both disease categories when classified by either % predicted FVC (P = 0.48) or % predicted D_L_CO (P = 0.29). There are no significant differences between mild and severe disease categories with respect to prior tobacco use. The diagnosis of IPF was confirmed by surgical lung biopsy in 50.7% of the study subjects (seeTables S1, S2, S3, and S4). A highly confident diagnosis of definite IPF was obtained in 90% of patients (64 of 71, Table S1, S2, S3, and S4). Five patients with mild disease by DLCO (≥ 65% predicted) failed to meet definite HRCT criteria for IPF due to minimal honeycombing, and are categorized as probable IPF since the predominate HRCT findings are sub-pleural, bilateral, bi-basilar reticulation, and traction bronchiectasis, and clinical features supported the diagnosis of IPF. Of these 5 cases, the mean age is 70 (range 63–75), 4 of the 5 cases are male, and is consistent with IPF.

**Table 1:** Clinical and demographic IPF variables categorized by FVC.

Variable	Characteristics	Mild IPF (N = 27)	Severe IPF (N = 13)	Controls (N = 27)
**% Predicted FVC**		85.0±8.1	42.5±6.6	NR
**Age**	Mean±SD	69.8±8.4	65.3±12.7	60.1±14.1
**Sex**	Male/Female	19/8	10/3	11/17
**Smoking Status**	Current	0	0	0
	Former	7	7	14
	Never	18	6	13
	Not Reported	2	0	0
				

Abbreviations: Idiopathic Pulmonary Fibrosis (IPF); Not Reported (NR); Diffusing Capacity for Carbon Monoxide (D_L_CO); Forced Vital Capacity (FVC).

**Table 2:** Clinical and demographic IPF variables categorized by D_L_CO.

Variable	Characteristics	Mild IPF (N = 16)	Severe IPF (N = 15)	Controls (N = 27)
**% Predicted D_L_CO**		77.1±11.9	27.4±5.3	NR
**Age**	Mean±SD	67.4±6.0	66.8±13.7	60.1±14.1
**Sex**	Male/Female	11/5	11/4	11/17
**Smoking Status**	Current	0	0	0
	Former	7	10	14
	Never	8	5	13
	Not Reported	1	0	0
				

Abbreviations: Idiopathic Pulmonary Fibrosis (IPF); Not Reported (NR); Diffusing Capacity for Carbon Monoxide (D_L_CO); Forced Vital Capacity (FVC).

### Identification of a Peripheral Blood Signature that Distinguishes Presence of Disease

**Figure 1:**
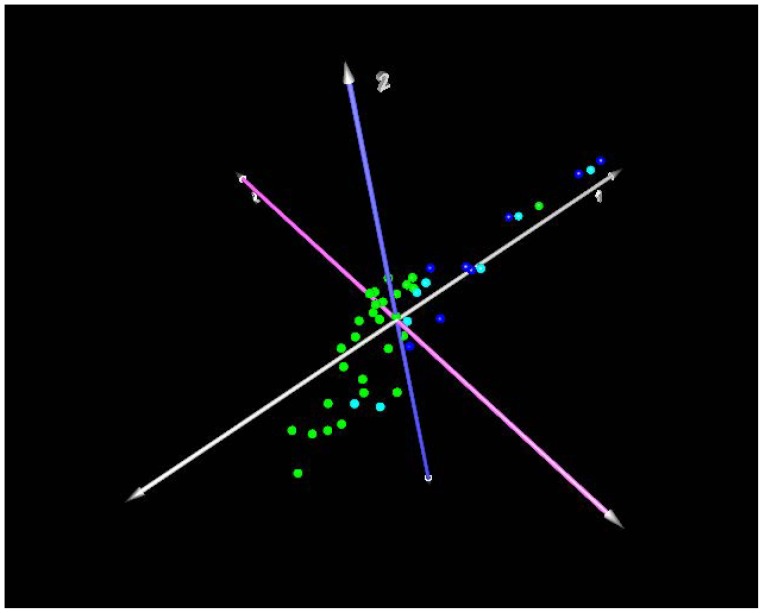
Principal component analysis of mild IPF cases (D_L_CO ≥65% predicted) compared to controls. Dark blue spheres: definite IPF; Cyan/light blue spheres: probable IPF; Green spheres: healthy controls. Axis labels: white-first principal component; blue-second principal component; lavender- third principal component. The majority of cases, both probable and definite, cluster along the first principal component. Two cases of probable IPF distribute with controls, and one control distributes with cases along the first principal component.

**Figure 2:**
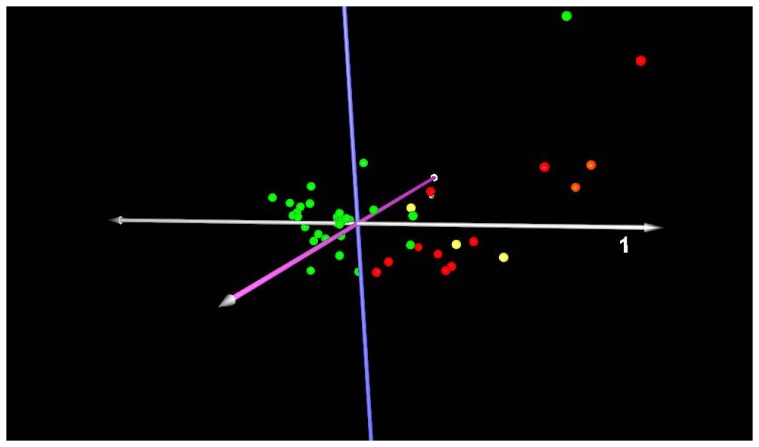
Principal component analysis of severe IPF (D_L_CO ≤35% predicted). Red spheres: definite IPF; Orange spheres: probable IPF; Yellow spheres: unclassifiable fibrosis; Green spheres: healthy controls. Axis labels: white-first principal component; blue-second principal component; lavender- third principal component. The majority of severe cases, both probable and definite, cluster along the first principal component. Three cases of unclassifiable fibrosis distribute with IPF cases.

Principal component analysis (PCA) was performed to determine outliers in the gene expression data based on disease categorization (mild or severe IPF vs. normal). The majority of mild cases (D_L_CO ≥65% predicted), both probable and definite, cluster along the first principal component (Figure 1). Two cases of probable IPF distribute with controls, and one control distributes with cases along the first principal component. Both of the probable cases that distributed with controls have very early disease, and they had mild disease on HRCT. The first case had a normal D_L_CO of 99% predicted, only a mildly reduced FVC of 71% predicted, and the second case case had a DLCO of 66% and FVC of 86% predicted. Severe cases of IPF (D_L_CO ≤35%), both probable and definite, distribute together along the first principal component (Figure 2). Three cases of advanced pulmonary fibrosis that by pathology review were deemed unclassifiable fibrotic lung disease due to advanced honeycomb lung but most consistent with IPFwere included in the analysis (Tables S1, S2, S3), and are similar to other cases of severe definite IPF. This demonstrates the potential of the peripheral blood molecular signature to diagnose patients when the interpretation of surgical lung biopsy is ambiguous. One control distributes with cases and is misclassified by PCA, and one control is not readily classified based on the first and second principal components. Thus, it is unlikely that the peripheral blood signature will achieve 100% accuracy to predict the presence of IPF.

Significant analysis of microarray (SAM) of the mild IPF cohort, when categorized by percent predicted D_L_CO (N = 16) compared to normal controls (N = 31) revealed 1,428 differentially-expressed transcripts (Table S6), and when categorized as severe IPF (N = 15) compared to normal controls (N = 31), 2,790 differentially-expressed transcripts (table S7) with <1% false discovery rate (FDR). Tables 3 and 4 list the differentially-expressed genes with at least a log_2_ 2 fold change in expression (While a few genes are in common, there are different sets of genes have at least a log_2_ 2-fold change in expression between mild cases compared to controls (table 3) compared to those that distinguish severe cases from control (table 4). Therefore, a molecular signature in the peripheral blood to detect IPF will be comprised of different genes depending on disease severity.

**Table 3:** Differentially-expressed genes that distinguish mild IPF from control.

Gene Symbol	Description	Fold Change‡
CEACAM4	carcinoembryonic antigen-related cell adhesion molecule 4	4.02
IL1R2	interleukin 1 receptor, type II	2.69
FCN1	ficolin (collagen/fibrinogen domain containing) 1	2.36
GRN	granulin	2.33
PTGIR	prostaglandin I2 (prostacyclin) receptor (IP)	2.30
HLA-B	major histocompatibility complex, class I, B	2.27
DYSF	dysferlin, limb girdle muscular dystrophy 2	2.25
LILRB3	leukocyte immunoglobulin-like receptor, subfamily B (3)	2.21
TALDO1	transaldolase 1	2.21
CXCR2	chemokine (C-X-C motif) receptor 2	2.19
FKBP5	FK506 binding protein 5	2.17
SORL1	sortilin-related receptor, L(DLR class) A repeats-containing	2.16
IMPDH1	IMP (inosine monophosphate) dehydrogenase 1	2.15
DAPK2	death-associated protein kinase 2	2.14
CA4	carbonic anhydrase IV	2.13
MMP9	matrix metallopeptidase 9 (gelatinase B)	2.11
PSAP	prosaposin	2.09
TUBA3D	tubulin, alpha 3d	−2.08
RPL24	ribosomal protein L24	−2.17
GPR78	G protein-coupled receptor 78	−2.68

‡ Significance analysis of microarrays (SAM) of IPF samples when categorized by percent predicted D_L_CO ≥65% [N = 16]. Differentially- expressed transcripts with <1% false discovery rate and > 2-fold change in expression are represented. Fold changes are expressed as log_2_ ratio. See supplementary tables for a complete list of differentially-expressed genes and corresponding accession numbers.

**Table 4:** Differentially-expressed genes that distinguish severe IPF from control.

Gene Symbol	Description	Fold Change
IL1R2	interleukin 1 receptor, type II	3.43
DEFA3	defensing, alpha 3, neutrophil specific	3.39
OLFM4	olfactomedin 4	3.39
MMP9	matrix metallopeptidase 9 (gelatinase B)	3.32
GRB10	growth factor receptor-bound protein 10	3.25
DEFA4	defensin, alpha 4, corticostatin	3.00
LTF	lactotransferrin	2.97
RAB8A	RAB8A, member RAS oncogene family	2.76
CTSG	cathepsin G	2.64
CAMP	cathelicidin antimicrobial peptide	2.64
CEACAMP8	carcinoembryonic antigen-related cell adhesion (8)	2.53
VSIG4	V-set and immunoglobulin domain containing 4	2.50
PGLYRP1	peptidoglycan recognition protein 1	2.45
FKBP5	FK506 binding protein 5	2.45
LOC151438	hypothetical protein LOC151438	2.43
ECHDC3	enoyl Coenzyme A hydratase domain containing 3	2.34
LOC100130890	similar to hCG2030844	−2.34
PRSS36	protease, serine, 36	−2.37
MCAT	malonyl CoA:ACP acyltransferase (mitochondrial)	−2.42
IGHM	immunoglobulin heavy constant mu	−3.0

† Significance analysis of microarrays (SAM) of IPF samples when categorized by percent predicted D_L_CO ≤35% [N = 15]. Differentially- expressed transcripts with <1% false discovery rate and ≥ 2-fold change in expression are represented. Fold changes are expressed as log_2_ ratio. See supplementary tables for a complete list of differentially-expressed genes and corresponding accession numbers.

### Identification of a Peripheral Blood Signature that Distinguishes Extent of Disease

**Figure 3:**
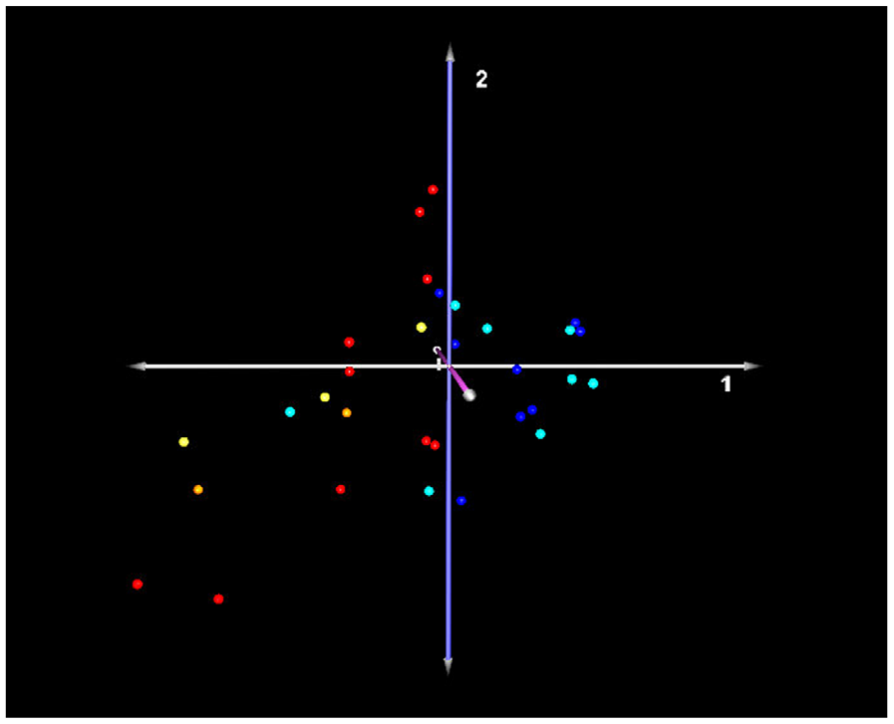
Principal component analysis of IPF samples grouped by extent of disease correlated with D_L_CO % predicted (mild ≥65%, severe ≤35%). Red spheres: definite IPF; Orange spheres: probable IPF; Yellow spheres: unclassifiable fibrosis;Blue spheres: mild IPF; Green spheres: severe cases of IPF. Axis labels: white-first principal component; blue-second principal component; lavender- third principal component.

Principal component analysis was performed to determine outliers in the data set based on severity of disease categorization. Results demonstrate that 1 severe IPF case appears to be clinically misclassified as a mild case of IPF, while other cases are correctly classified as mild or severe (Figure 3). Significance analysis of microarrays (SAM) of IPF samples, when categorized by percent predicted D_L_CO (D_L_CO ≥65% [N = 16] and D_L_CO ≤35% [N = 15]), demonstrated 13 differentially-expressed transcripts with less than a 5% false discovery rate. Table 5 lists all differentially-expressed genes found between mild and severe cases of IPF categorized by D_L_CO. When using a FDR of ≤1%, only defensin A3 (DEFA3) is differentially-expressed between both mild or severe cases compared to controls. SAM revealed no differentially-expressed transcripts with less than a 5% false discovery rate between peripheral blood samples when IPF patients were categorized by percent predicted FVC (N = 27 and N = 13, data not shown).

**Table 5:** Differentially-expressed genes between mild and severe cases of IPF.

Symbol	Entrez Gene Name	Probe ID	Accession Number	Fold Change	Location
**CAMP**	cathelicidin antimicrobial peptide	A_23_P253791	NM_004345	2.591	Cytoplasm
**CEACAM6 (includes EG:4680)**	carcinoembryonic antigen-related cell adhesion molecule 6	A_23_P421483	BC005008	2.353	Plasma Membrane
**CTSG**	cathepsin G	A_23_P140384	NM_001911	2.703	Cytoplasm
**DEFA3 (includes EG:1668)**	defensin, alpha3, neutrophil-specific	A_23_P31816	NM_005217	2.379	Extracellular Space
**DEFA4 (includes EG:1669)**	defensin, alpha 4, corticostatin	A_23_P326080	NM_001925	3.713	Extracellular Space
**OLFM4**	olfactomedin 4	A_24_P181254	NM_006418	3.807	unknown
**HLTF**	helicase-like transcription factor	A32_P210798	BF513730	1.413	unknown
**PACSIN1**	protein kinase C and casein kinase substrate in neurons 1	A_23_P258088	NM_020804	−1.511	Cytoplasm
**FLJ11710**	hypothetical protein FLJ11710	A_23_P3921	AK021772	−1.798	unknown
**GABBR1**	gamma-aminobutyric acid (GABA) B receptor, 1	A_23_P93302	NM_001470	−1.471	Plasma Membrane
**IGHM**	immunoglobulin heavy constant mu	A_24_P417352	BX161420	−2.451	Plasma Membrane
**unknown**	Unknown	A_23_P91743	unknown	−1.884	unknown
**unknown**	Unknown	A_24_P481375	AK021668	−1.706	unknown

Alpha defensins activation pathway. Solid lines (direct relationship); Dashed lines (indirect relationship).

### Peripheral Blood Signature Disease Progression Analysis

The general comparison analysis tool of the Ingenuity Pathway Analysis (IPA) software was utilized to identify the intersection or common differentially-expressed transcripts between normal and mild IPF, normal and severe IPF, and mild versus severe IPF when classified by D_L_CO for the purpose of discovery common potential biomarkers. This analysis showed that at FDR <5% only 2 differentially expressed transcripts, A3 (DEFA3) and hypothetical protein FLJ11710 , were differentially-expressed between control vs mild, mild vs severe, and control vs severe disease (D_L_CO ≤35%). There is up-regulation in DEFA3 expression compared to controls for severe IPF, while hypothetical protein FLJ11710 demonstrates a down regulation compared to controls for severe IPF. Between the mild and severe IPF cohort , and between normal and severe disease, there is up-regulation of several other host defense genes including defensin A4 (DEFA4), cathelicidin antimicrobial peptide (CAMP), cathespin G (CTSG), and down-regulation of immunoglobulin heavy chain constant mu (IGHM). The greatest increase found distinguishing controls from severe disease, and mild from severe disease is olfactinmedin 4 (OLFM4). We randomly selected 4 of the 13 differentially-expressed genes for qPCR validation, and qPCR confirmed the differential expression of these genes identified by microarray experiments (Table S8).

**Figure 4:**
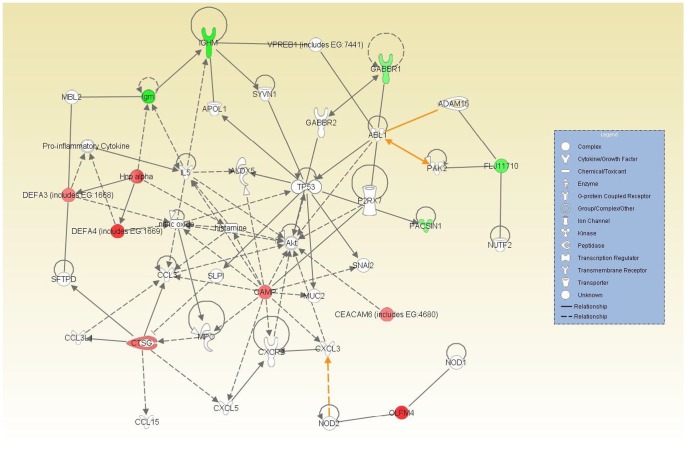
Overlaid networks and associated pathway analysis . Solid lines (direct relationship); Dashed lines (indirect relationship); Red filled (up-regulation); and Green filled (down-regulation).

**Figure 5:**
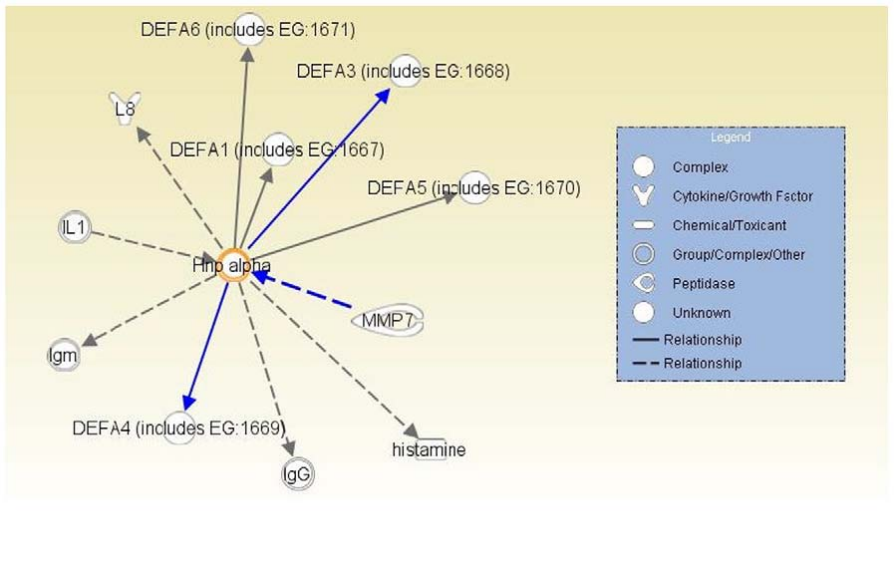
Alpha defensins activation pathway. Solid lines (direct relationship); Dashed lines (indirect relationship).

We also subjected the list of 13 differentially expressed genes based on D_L_CO (5%FDR, Table 5) to a functional analysis utilizing the Ingenuity Pathway Analysis (IPA) software. The functional analysis tool also calculates a significance value that is a measure for the likelihood that the association between a set of genes and a given process is due to random chance. Ten of the 13 differentially-expressed genes had annotations representing a gene, protein or chemical that was able to be mapped to an associated network. The associated network functions (Table S5) included: 1) inflammatory response (P<0.05), 2) cellular movement (P<0.05), 3) immune trafficking, 4) genetic disorder (P<0.05); and 4) cell-to-cell signaling (P<0.05). Figure 4 shows an overlay of all three associated networks illustrating both direct and indirect relationship pathways of the differentially-expressed genes. Finally, given that more than one α-defensin and other host defense-related proteins that distinguish normal, mild, and severe disease, we analyzed pathway interactions with α-defensins (Figure 5), and note an interaction with metalloproteinase 7 (MMP7) which activates α-defensins by proteolytic cleavage.

## Discussion

This is the first study to investigate the IPF peripheral blood transcriptome based on disease severity defined by lung functional parameters. Our findings provide evidence that the peripheral blood transcriptome can be used to develop signatures that identify the presence and disease extent of IPF. We demonstrate a composite signature that distinguishes normal controls from IPF, 13 genes that distinguish mild from severe IPF utilizing D_L_CO values. Importantly, when a stringent false discovery rate of ≤1% is used, the genes that demonstrate the greatest differential-expression between control and mild disease, and control and severe disease are different. These data indicate that molecular signatures from the peripheral blood transcriptome that are intended to predict the presence of IPF will need to be take into account disease severity. There was differential-expressionof several inflammatory response and immune trafficking genes including α-defensins that are proteolytic substrates of MMP7. MMP7 has previously been shown to be over-expressed in IPF lung and serum, and its increased expression is associated with decreased survival [20]. These findings suggest that decreases in D_L_CO in IPF may be related to immune trafficking genes such as α-defensins.

We have identified that the peripheral blood transcriptome has the potential to discriminate controls from mild or severe IPF, and distinguish mild from severe disease using a cohort of both sporadic and familial IPF. Our previous gene expression studies indicate sporadic and familial IPF have similar gene expression patterns in lung [21]. Additionally, we have previously identified a promoter polymorphism in MUC5B that increase the risk of developing either sporadic or familial IPF [22]. Consequently, we believe both sporadic and familial IPF can be considered to be more similar than different, and justifies combining these in the current analysis.

Recent studies using plasma or serum protein markers have identified several proteins that might indicate disease presence or survival. Reduced survival has been associated with elevated serum levels of mucin 1 (MUC1/KL6) [23], chemokine (C-C motif) ligand 18 (CCL-18) [24], or surfactant protein A [25]. Rosas et al used a multi-analyte approach to screen a panel of 49 serum proteins and identified a combinatorial signature of 5 proteins that distinguished IPF patients from controls. MMP7 and MMP1 were the main components of this signature [15]. In their study, higher levels of MMP7 appeared to be associated with more severe disease. Most recently, Richards et al identified 140 patients with IPF and then validated in another 101 patients increased serum protein levels of MMP-7, ICAM-1, IL-8, VCAM-1, and S100A12 to be associated with decreased survival (XX). These studiesand ours demonstrate the emerging trend to identify relevant novel diagnostic and prognostic biomarkers for IPF in blood. There are no studies that evaluate on a genome-wide basis the peripheral blood transcriptome of IPF which relate to diagnostic or prognostic signatures.

Four of the 13 genes up-regulated in the current study have functions pertaining to host defense. This raises the question of an association of advanced disease and sub-clinical infection, or host-microbe interactions. All of these patients were stable when blood was drawn without overt signs of infection. Viral infection has been put forth as a possible trigger of acute exacerbations of IPF [12]. Two of the 13 genes are α-defensins which are small, cationic, cysteine-rich antimicrobial peptides that have important roles in host defense against bacteria, fungi and enveloped viruses [26]. In humans, α-defensins 1–4 are primarily found in neutrophils, and β-defensins are found in the epithelia of mucosal surfaces. Both are up-regulated by bacterial and viral infection [27], [28]. α-defensins are synthesized as inactive precursors consisting of 29–42 amino acid residues and are activated by proteolytic cleavage both by MMP7 [29], [30]. α-defensin levels in bronchoalveolar lavage and/or plasma are increased in fibrotic lung diseases, and significant amounts of α-defensins can be found outside neutrophils in fibroblastic foci in the lungs of patients with IPF [31]. Increased α-defensins levels are detected in the lung and blood of patients with acute exacerbations of IPF [32]. Elevated serum MMP-7 protein distinguish IPF from other types of diffuse parenchymal lung disease, and higher serum levels of MMP-7 in patients with IPF is associated with worse lung function [33]. MMP-7 expression is also up-regulated in lungs of patients with IPF [34]. In inflammatory lung disease complicated by fibroproliferation, it has been reported that α-defensins may contribute to the fibrotic response [12], [35], [36]. These data support the hypothesis that elevated levels of α-defensins may be an important substrate for MMP-7, and this interaction may be related to worsening physiologic function (D_L_CO).

Differential-expression analysis demonstrates up-regulation of the cathelicidin antimicrobial peptide (CAMP) between the severe IPF subgroup compared to controls, and between mild and severe disease groups. Cathelicidin is an antimicrobial protein of the innate immune system stored in peroxidase-negative granules of neutrophils [37]. CAMP is widely distributed, expressed in lung tissue, and detected in peripheral blood, plasma as well as bronchoalveolar lavage fluid (BAL) [38]. CAMP is another molecule of innate immunity that distinguishes mild from severe disease. While hypothetical protein FLJ11710 was down regulated in IPF groups compared to controls, little is known about its molecular functionality. CEACAM proteins have previously been shown to bind gram negative bacteria and are also over-expressed in lung cancers, associated with anti-apoptotic properties and tumor metastases. The CEACAM-1 gene encodes for glycosylated, glycosylphosphatidylinositol (GPI) anchored proteins that are expressed in alveolar epithelial cells [39]. Olfactomedin 4 is expressed primarily in bone marrow cells, but also in prostate, small intestine, colon, and stomach, and is upregulated in cancers of the stomach, colon, breast, and lung [40], [41]. Olfactomedin 4 appears to promote S-phase transition, and is a marker of intestinal stem cells [42]. An explanation for the increased expression of olfactomedin 4 in the peripheral blood of IPF patients, and its ability to distinguish mild from severe disease, is difficult.

There are limitations to this study. Given the minimal disease burden and lack of clinical symptoms, five cases of mild IPF were not appropriate candidates for surgical lung biopsy, and therefore did not have surgical tissue. Due to the minimal disease burden, they did not meet HRCT criteria of definite IPF according to the 2011 ATS/ERS/JRS/ALAT consensus statement. However, in the five cases the mean age was 70, and 4 cases were male supporting the diagnosis of IPF. Furthermore, these cases cluster with definite mild cases along the principal components (figure 1). Therefore, while these five cases of do not meet definite disease by current criteria, they very likely represent an early stage of IPF when all the definite criteria of disease cannot be met. Furthermore, the fact that these cases cluster with definite cases based on the peripheral blood signature demonstrates the feasibility of the peripheral blood gene expression signature as a diagnostic tool for early stage disease when HRCT findings are often ambiguous. There is no longitudinal data on the early cases to determine disease progression or other clinical variables. We have identified a signature that distinguishes disease extent (% predicted D_L_CO), but not when correlated with by FVC % predicted. The reason for this difference is unclear. FVC is traditionally used to stage the severity of pulmonary fibrosis. However, it has been recognized that a single measurement of the FVC is not useful for predicting disease progression or mortality of IPF [43]. In contrast, a 10 % decrease in FVC is associated with increased mortality in IPF [43]. Had we been able to obtain serial measurements of FVC, then perhaps we might have found an association with peripheral blood gene expression, disease severity, and FVC. In contrast, a single measurement of D_L_CO <50% predicts an increased mortality [43]. Therefore, it is possible that the peripheral blood signatures described correlate with disease progression as measured by a single D_L_CO% predicted but not a single FVC% predicted measurement due to the better performance of a single measurement of D_L_CO compared to FVC. A lack of correlation between FVC and severity of IPF can be due to concomitant emphysema, however review of the subject’s HRCT in this study does not demonstrate significant amounts of emphysema. It is possible the signature is a consequence of impaired oxygen transport, but this seems unlikely since two of the thirteen genes also distinguish controls from mild disease where oxygen transport is not a factor. Also, none of the 13 genes have functions related to oxygen transport, aerobic or anaerobic metabolism, or other pathways that might be implicated in the presence of hypoxemia or impaired oxygenation.

In summary, our results demonstrate that the peripheral blood transcriptome can potentially distinguish extent of disease in individuals with IPF when samples are correlated with percent predicted D_L_CO, and distinguish IPF patients from normal. Our data is consistent with a role for MMP-7 interacting with α-defensins, and increased expression of host defense proteins in the peripheral blood being associated with deterioration of D_L_CO in IPF. The ability to use a peripheral blood biomarker to monitor disease progression for IPF could have a substantial impact on the diagnosis, treatment, and management of this disease, and perhaps be generally applicable to other subtypes of idiopathic interstitial pneumonias.

## Supporting Information

Table S1 **†Definite IPF without surgical lung biopsy is defined by supporting clinical information and HRCT demonstrating sub-pleural and bibasilar predominate reticulation, honeycombing, and traction bronchiectasis without atypical features such as nodules, predominate ground glass opacities, pleural plaques, air-trapping, or lymphadenopathy.** ‡Probable IPF without surgical lung biopsy is defined by supporting clinical information and HRCT demonstrating sub-pleural and bibasilar predominate reticulation, traction bronchiectasis without bilateral honeycombing, and without atypical features outlined above. Surgical lung biopsy (SLBx): definite IPF is defined as usual interstitial pneumonia requiring spatial and temporal heterogeneity; subpleurally accentuated microscopic honeycombing, fibroblastic foci without significant parenchymal, airway, or pleural mononuclear inflammation; definite IPF is also advanced honeycombing on lung biopsy with clinical and radiologic features supporting IPF. n/a is not available.
(DOCX)

Table S2 **†Definite IPF without surgical lung biopsy is defined by supporting clinical information and HRCT demonstrating sub-pleural and bibasilar predominate reticulation, honeycombing, and traction bronchiectasis without atypical features such as nodules, predominate ground glass opacities, pleural plaques, air-trapping, or lymphadenopathy.** ‡Probable IPF without surgical lung biopsy is defined by supporting clinical information and HRCT demonstrating sub-pleural and bibasilar predominate reticulation, traction bronchiectasis without bilateral honeycombing, and without atypical features outlined above. Surgical lung biopsy (SLBx) :definite IPF is defined as usual interstitial pneumonia requiring spatial and temporal heterogeneity; subpleurally accentuated microscopic honeycombing, fibroblastic foci without significant parenchymal, airway, or pleural mononuclear inflammation; definite IPF is also advanced honeycombing on lung biopsy with clinical and radiologic features supporting IPF. n/a is not available.
(DOCX)

Table S3 **†Definite IPF without surgical lung biopsy is defined by supporting clinical information and HRCT demonstrating sub-pleural and bibasilar predominate reticulation, honeycombing, and traction bronchiectasis without atypical features such as nodules, predominate ground glass opacities, pleural plaques, air-trapping, or lymphadenopathy.** ‡Probable IPF without surgical lung biopsy is defined by supporting clinical information and HRCT demonstrating sub-pleural and bibasilar predominate reticulation, traction bronchiectasis without bilateral honeycombing, and without atypical features outlined above. Surgical lung biopsy (SLBx) :definite IPF is defined as usual interstitial pneumonia requiring spatial and temporal heterogeneity; subpleurally accentuated microscopic honeycombing, fibroblastic foci without significant parenchymal, airway, or pleural mononuclear inflammation; definite IPF is also advanced honeycombing on lung biopsy with clinical and radiologic features supporting IPF. n/a is not available.
(DOCX)

Table S4 **†Definite IPF without surgical lung biopsy is defined by supporting clinical information and HRCT demonstrating sub-pleural and bibasilar predominate reticulation, honeycombing, and traction bronchiectasis without atypical features such as nodules, predominate ground glass opacities, pleural plaques, air-trapping, or lymphadenopathy.** ‡Probable IPF without surgical lung biopsy is defined by supporting clinical information and HRCT demonstrating sub-pleural and bibasilar predominate reticulation, traction bronchiectasis without bilateral honeycombing, and without atypical features outlined above. Surgical lung biopsy (SLBx): definite IPF is defined as usual interstitial pneumonia requiring spatial and temporal heterogeneity; subpleurally accentuated microscopic honeycombing, fibroblastic foci without significant parenchymal, airway, or pleural mononuclear inflammation; definite IPF is also advanced honeycombing on lung biopsy with clinical and radiologic features supporting IPF. n/a is not available.
(DOCX)

Table S5 **Canonical pathway analysis using Ingenuity Pathway Analysis™ tool to identify associated networks.** Network functions with their respective P values and the number of differentially-expressed genes identified are presented.
(DOCX)

Table S6 **Differentially-expressed genes between controls and mild IPF (D_L_CO >65%) using SAM at FDR of <1%.** Agilent probe identifier, RefSeq number, and Entrez geneID number are presented. Fold change is log_2._ FDR is false discover rate (%).
(XLSX)

Table S7 **Differentially-expressed genes between controls and severe IPF (D_L_CO <35%) using SAM at FDR of <1%.** Agilent probe identifier, RefSeq number, and Entrez geneID number are presented. Fold change is log_2._ FDR is false discover rate (%).
(XLSX)

Table S8 **Validation of selected differentially-expressed genes by Reatime™ PCR.** Primers utilized for PCR and differential expression by Realtime PCR using ΔΔ Ct method.
(XLSX)
