# Supplementary material for: The Peripheral Blood Transcriptome Identifies the Presence and Extent of Disease in Idiopathic Pulmonary Fibrosis
Source: PLoS One. 2012 Jun 22;7(6):e37708. doi: 10.1371/journal.pone.0037708 (PMC3382229; doi:10.1371/journal.pone.0037708)
Supplement: Table S1 — †Definite IPF without surgical lung biopsy is defined by supporting clinical information and HRCT demonstrating sub-pleural and bibasilar predominate reticulation, honeycombing, and traction bronchiectasis without atypical features such as nodules, predominate ground glass opacities, pleural plaques, air-trapping, or lymphadenopathy. ‡Probable IPF without surgical lung biopsy is defined by supporting clinical information and HRCT demonstrating sub-pleural and bibasilar predominate reticulation, traction bronchiectasis without bilateral honeycombing, and without atypical features outlined above. Surgical lung biopsy (SLBx): definite IPF is defined as usual interstitial pneumonia requiring spatial and temporal heterogeneity; subpleurally accentuated microscopic honeycombing, fibroblastic foci without significant parenchymal, airway, or pleural mononuclear inflammation; definite IPF is also advanced honeycombing on lung biopsy with clinical and radiologic features supporting IPF. n/a is not available. (DOCX) [file pone.0037708.s001.docx]

| Table S1: Phenotype Data of Mild Disease Group Categorized by DLCO > 65% | | | | | | | |
| --- | --- | --- | --- | --- | --- | --- | --- |
| **D_L_CO**  (%) | **FVC**  **(%)** | **Age**  **(yr.)** | **Smoking Status** | **Gender** | **SLB** | **Dx** | **Certainty** |
| 65 | 53 | 62 | former | M | Y | IPF | Definite |
| 65 | 70 | 61 | former | M | Y | IPF | Definite |
| 66 | 94 | 75 | never | F | N | IPF | †Definite |
| 66 | 77 | 71 | never | M | N | IPF | Definite |
| 66 | 81 | 68 | former | M | Y | IPF | Definite |
| 69 | 91 | 73 | never | M | N | IPF | Definite |
| 71 | n/a | 75 | former | F | N | IPF | ‡Probable |
| 75 | 86 | 68 | never | F | Y | IPF | Definite |
| 77 | 83 | 67 | former | M | N | IPF | Definite |
| 78 | n/a | 63 | former | F | Y | IPF | Definite |
| 79 | 76 | 54 | never | M | N | IPF | Probable |
| 83 | 92 | 63 | never | M | Y | IPF | Definite |
| 85 | 77 | 75 | never | M | N | IPF | Probable |
| 87 | n/a | 66 | never | F | Y | IPF | Definite |
| 99 | 71 | 65 | never | M | N | IPF | Probable |
| 103 | 111 | 72 | former | M | N | IPF | Probable |
