# Supplementary material for: The Peripheral Blood Transcriptome Identifies the Presence and Extent of Disease in Idiopathic Pulmonary Fibrosis
Source: PLoS One. 2012 Jun 22;7(6):e37708. doi: 10.1371/journal.pone.0037708 (PMC3382229; doi:10.1371/journal.pone.0037708)
Supplement: Table S3 — †Definite IPF without surgical lung biopsy is defined by supporting clinical information and HRCT demonstrating sub-pleural and bibasilar predominate reticulation, honeycombing, and traction bronchiectasis without atypical features such as nodules, predominate ground glass opacities, pleural plaques, air-trapping, or lymphadenopathy. ‡Probable IPF without surgical lung biopsy is defined by supporting clinical information and HRCT demonstrating sub-pleural and bibasilar predominate reticulation, traction bronchiectasis without bilateral honeycombing, and without atypical features outlined above. Surgical lung biopsy (SLBx) :definite IPF is defined as usual interstitial pneumonia requiring spatial and temporal heterogeneity; subpleurally accentuated microscopic honeycombing, fibroblastic foci without significant parenchymal, airway, or pleural mononuclear inflammation; definite IPF is also advanced honeycombing on lung biopsy with clinical and radiologic features supporting IPF. n/a is not available. (DOCX) [file pone.0037708.s003.docx]

| Table S3: Phenotype Data of Mild Disease Group Categorized by FVC >75% | | | | | | | | |
| --- | --- | --- | --- | --- | --- | --- | --- | --- |
| **D_L_CO (%)** | **FVC (%)** | **Age (Yr.)** | **Smoking Status** | **Gender** | **SLBx** | **Dx** | **Certainty** |  |
| 79 | 76 | 54 | Never | M | Y | IPF | Definite |  |
| n/a | 76 | 59 | Former | M | N | IPF | †Definite |  |
| 85 | 77 | 75 | Former | M | N | IPF | Definite |  |
| 54 | 77 | 84 | Former | M | N | IPF | Definite |  |
| 66 | 77 | 71 | Never | M | N | IPF | Definite |  |
| n/a | 79 | 65 | Former | F | Y | IPF | Definite |  |
| 62 | 80 | 77 | Former | M | N | IPF | ‡Probable |  |
| n/a | 80 | 78 | Former | M | Y | IPF | Definite |  |
| n/a | 81 | 81.2 | Former | M | N | IPF | Definite |  |
| 38 | 81 | 63 | Former | M | Y | IPF | Definite |  |
| 47 | 81 | 84 | Former | F | N | IPF | Definite |  |
| 66 | 81 | 68 | Former | M | Y | IPF | Definite |  |
| 62 | 82 | 64 | Former | F | N | IPF | Definite |  |
| 77 | 83 | 67 | Former | M | N | IPF | Definite |  |
| n/a | 83 | 71 | Never | F | Y | IPF | Definite |  |
| 42 | 84 | 67.6 | n/a | M | Y | IPF | Definite |  |
| 60 | 86 | 71 | Former | M | Y | IPF | Definite |  |
| 75 | 86 | 68 | Never | F | Y | IPF | Definite |  |
| n/a | 87 | 51 | Never | M | Y | IPF | Definite |  |
| n/a | 90 | 71.1 | Former | F | N | IPF | Definite |  |
| 61 | 91 | 70 | Never | M | Y | IPF | Definite |  |
| 69 | 91 | 73 | Never | M | N | IPF | Definite |  |
| 83 | 92 | 62.7 | Never | M | Y | IPF | Definite |  |
| 66 | 94 | 75.4 | n/a | F | N | IPF | Definite |  |
| 59 | 101 | 81 | Former | M | N | IPF | Definite |  |
| 103 | 111 | 72 | Former | M | N | IPF | Definite |  |
| n/a | 88 | 59 | Former | F | Y | IPF | Definite |  |
