# Supplementary material for: The Peripheral Blood Transcriptome Identifies the Presence and Extent of Disease in Idiopathic Pulmonary Fibrosis
Source: PLoS One. 2012 Jun 22;7(6):e37708. doi: 10.1371/journal.pone.0037708 (PMC3382229; doi:10.1371/journal.pone.0037708)
Supplement: Table S4 — †Definite IPF without surgical lung biopsy is defined by supporting clinical information and HRCT demonstrating sub-pleural and bibasilar predominate reticulation, honeycombing, and traction bronchiectasis without atypical features such as nodules, predominate ground glass opacities, pleural plaques, air-trapping, or lymphadenopathy. ‡Probable IPF without surgical lung biopsy is defined by supporting clinical information and HRCT demonstrating sub-pleural and bibasilar predominate reticulation, traction bronchiectasis without bilateral honeycombing, and without atypical features outlined above. Surgical lung biopsy (SLBx): definite IPF is defined as usual interstitial pneumonia requiring spatial and temporal heterogeneity; subpleurally accentuated microscopic honeycombing, fibroblastic foci without significant parenchymal, airway, or pleural mononuclear inflammation; definite IPF is also advanced honeycombing on lung biopsy with clinical and radiologic features supporting IPF. n/a is not available. (DOCX) [file pone.0037708.s004.docx]

| Table S4: Phenotype Data of Severe Disease Group Categorized by FVC < 50% | | | | | | | | | | | |
| --- | --- | --- | --- | --- | --- | --- | --- | --- | --- | --- | --- |
| **D_L_CO**  **(%)** | **FVC**  **(%)** | **Age**  **(Yr.)** | **Smoking Status** | | **Gender** | | **SLBx** | **Dx** | | **Certainty** |  |
| 18 | 26 | 35 | Never | F | | Y | | IPF | Definite | |  |
| 19 | 37 | 70 | Never | M | | Y | | IPF | Definite | |  |
| 30 | 37 | 44 | Never | M | | Y | | IPF | Definite | |  |
| 19 | 41 | 76 | Former | M | | Y | | IPF | Definite | |  |
| 24 | 42 | 64 | Former | F | | Y | | IPF | Definite | |  |
| 25 | 43 | 74 | Former | M | | N | | IPF | Definite | |  |
| n/a | 43 | 74 | Never | M | | Y | | IPF | Definite | |  |
| 31 | 44 | 79 | Never | M | | N | | IPF | Definite | |  |
| 29 | 45 | 68 | Former | F | | N | | IPF | Definite | |  |
| 24 | 45 | 73 | Never | M | | Y | | IPF | Definite | |  |
| 47 | 50 | 63 | Former | M | | Y | | IPF | Definite | |  |
| n/a | 50 | 68 | Former | M | | N | | IPF | Definite | |  |
| 28 | 50 | 62 | Former | M | | Y | | IPF | Definite | |  |
