# Supplementary material for: The Peripheral Blood Transcriptome Identifies the Presence and Extent of Disease in Idiopathic Pulmonary Fibrosis
Source: PLoS One. 2012 Jun 22;7(6):e37708. doi: 10.1371/journal.pone.0037708 (PMC3382229; doi:10.1371/journal.pone.0037708)
Supplement: Table S5 — Canonical pathway analysis using Ingenuity Pathway Analysis™ tool to identify associated networks. Network functions with their respective P values and the number of differentially-expressed genes identified are presented. (DOCX) [file pone.0037708.s005.docx]

| **Table S5. p-value ranges for associated network bio-functions** | | |
| --- | --- | --- |
|  |  |  |
| **Function** | **p-Value Range** | **# of Molecules** |
| Inflammatory Response | 1.79E^-4 - 3.94E^-2 | 4 |
| Cellular Movement | 9.39E^-5 - 3.94E^-2 | 3 |
| Immune Trafficking | 1.23E^-4 - 3.94E^-2 | 4 |
| Genetic disorder | 1.04E^-3 - 4.29E^-2 | 4 |
| Cell-to-cell signaling | 6.07E^-4 - 4.17E^-2 | 5 |
